# Computed tomography findings in bilateral perinephric lymphangiomatosis

**DOI:** 10.4103/0974-7796.62922

**Published:** 2010

**Authors:** Aijaz Hakeem, Tariq A Gojwari, Sheikh Reyaz, Shubana Rasool, Hakim Shafi, Shahida Mufti

**Affiliations:** Department of Radio-diagnosis, SK Institute of Medical Sciences and Government Medical College, Srinagar, Kashmir - 190 011, India; 1 Department of Gynecology, SK Institute of Medical Sciences and Government Medical College, Srinagar, Kashmir - 190 011, India; 2 Department of Medicine, SK Institute of Medical Sciences and Government Medical College, Srinagar, Kashmir - 190 011, India

**Keywords:** CT, perinephric collection, renal lymphangiomatosis

## Abstract

Perinephric lymphangioma is rare disorder that may be confused with various forms of renal cystic diseases and urinomas. In this disorder a developmental malformation results in failure of developing lymphatic tissue to establish normal communication with the rest of lymphatic system. Once there is restricted drainage of lymphatic fluid the lymphatic channels dilate to form cystic masses that may be unilocular or multilocular and may be seen unilaterally or bilaterally .This condition presents with various signs and symptoms or can be just an incidental finding which in presence of misleading clinical history may be confused with other diseases. CT scan with delayed cuts and USG guided aspiration with biochemical analysis of fluid will help us in arriving to final diagnosis.

## INTRODUCTION

Perinephric lymphangioma is rare disorder that may be confused with various forms of renal cystic diseases and urinomas. In this disorder a developmental malformation results in failure of developing lymphatic tissue to establish normal communication with the rest of lymphatic system. Once there is restricted drainage of lymphatic fluid the lymphatic channels dilate to form cystic masses that may be unilocular or multilocular and may be seen unilaterally or bilaterally .This condition presents with various signs and symptoms or can be just an incidental finding which in presence of misleading clinical history may be confused with other diseases. CT scan with delayed cuts and USG guided aspiration with biochemical analysis of fluid will help us in arriving to final diagnosis.

## CASE REPORT

**Figure 1:**
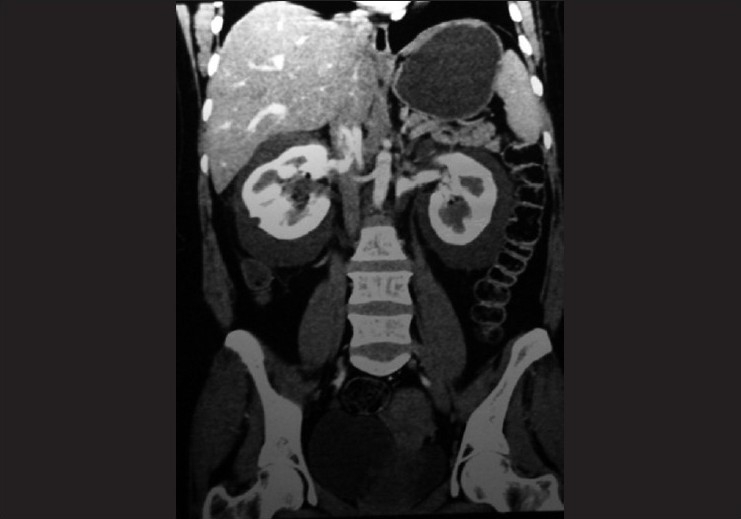
Coronal CT image showing bilateral perinephric collections with otherwise normal kidneys

**Figure 2:**
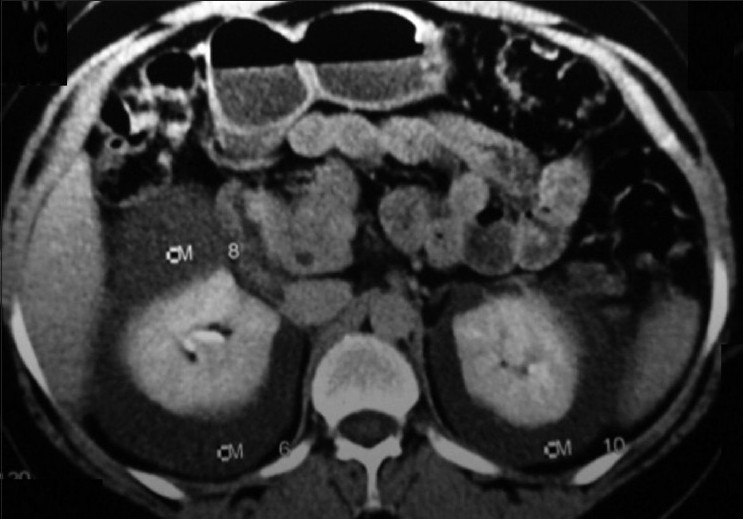
Axial CT scan showing fluid attenuation perinephric collections

A 50-year-old postmenopausal female with multiple episodes of urine retention and uterine prolapse consulted the department of gynecology. On physical examination, she was well built, apyrexic with normal blood pressure and pulse of 78 beats per minute. Her systemic examination was unremarkable. Local genital examination revealed grade-4 uterine prolapse with decubitus cervical ulcer, grade-2 rectocele, and grade-3 cystocele. Before the patient was planned for vaginal hysterectomy with colpoperineorrhaphy, a preoperative ultrasonographic evaluation was performed at the department of radiodiagnosis. The ultrasonography (done on Siemens Adara Sonoline ultrasound machine) revealed bilateral septated perinephric fluid collections with otherwise normal study. With ultrasonographic features and clinical history of urine retention, a provisional diagnosis of perinephric urinoma was made; however for confirmation, intraveous urography and a contrast enhanced CT scan of the abdomen were performed. The result of intravenous urography was unremarkable. A subsequent CT scan (Siemen's sensation 64 slice CT) showed bilateral perinephric fluid collection of attenuation 5–10 HU [Figure 1]. No other abnormality was detected on CT scan. Delayed scans failed to reveal contrast extravazation into perinephric space or any increase in attenuation of fluid in this space [Figure 2], thus ruling out any communication with pelvicalyceal system. An ultrasound-guided aspiration of fluid was also done which again failed to show the urinary nature of the fluid. On biochemical analysis, the fluid was aseptic and chylous with good number of lymphocytes, confirming the perinephric collection as lymphatic fluid and disorder as incidental perinephric lymphangiomatosis. The patient successfully underwent hysterectomy with colpoperineorrhaphy, with postoperative period being uneventful. Follow-up after four months with ultrasonogram did not show any increase in size of perinephric lymphangiomas.

## DISCUSSION

Renal lymphangiomatosis is an extremely rare developmental disorder of lymphatic system surrounding kidneys. They can occur in any location where lymphatics are normally present. The frequency of lymphangiomas are 75% in head and neck, 20% in axillary region, and 5% at other less common sites.[1] Retroperitoneal lymphangiomas account for approximately 1% of all lymphangiomas, with 185 cases identified in a review of international literature.[2] The reason for the development of this abnormality is failure of drainage of lymphatics into retroperitoneal lymphatic system.[3] It may be asymptomatic or may present with nonspecific symptomatology. The commonest complaints include flank pain, abdominal distension, hematuria, hypertension, fever, and rarely impaired renal function.[3,4] This condition is found in children as well as in adults.[5,6] Familial association has also been described.[7] One of the main features of retroperitoneal lymphangioma is that the mass is generally of water density on CT or MRI.[8] Prior to the advent of CT scan, the diagnosis was usually made at the time of exploratory laparotomy or after nephrectomy. On CT scan, renal lymphangiectasia appears as a well contained, fluid attenuating collections in the peripelvic or perinephric space with or without demonstrable septations with normal renal parenchyma.[1,9] When intrarenal lymphatics are solely involved, renal lymphangiectasia may present as simple enlargement of the kidney or even as a solid mass without evidence of any cystic lesion.[10] On MRI, they are hypointense on T1W images and hyperintense on T2W images.[11] The diagnosis of renal lymphangiectasia can be confirmed with needle aspiration of chylous fluid from the perinephric fluid collections.[12] However, the ultrasonographic and CT findings are characteristic for this disease and allow the diagnosis to be made confidently.[13,14] Treatment is not usually necessary. Complicated cases may be treated with nephrectomy, percutaneous drainage, or marsupialization.[12] Surgery is often required for symptom control or diagnosis.[15] Recurrence of symptoms with incomplete excision is possible.[16] Injection of sclerosants such as alcohol and bleomycin into lymphangiomas has been described in the literature in nonsurgical candidates. However, induration of the cyst and infection often complicate these procedures.[17] Complications of undiagnosed/untreated cases include hematuria, ascites, hypertension, renal vein thrombosis, and impairment of renal function.[18,19]

## CONCLUSION

Perinephric lymphangiomas can present with a number of nonspecific symptoms, and high blood pressure however can present as an incidental finding for which nothing needs to be done till patient remains asymptomatic. The close differential diagnosis is perinephric urinoma which can be ruled out with a contrast enhanced CT scan with delayed cuts, however, biochemical examination of aspirated fluid is also vital in reaching a final diagnosis.
